# Significance of Population Health Knowledge in the Education of the Undergraduate Workforce for Careers in the Health Care Sector

**DOI:** 10.3389/fpubh.2014.00212

**Published:** 2014-10-27

**Authors:** Stephen A. Martin

**Affiliations:** ^1^Association for Community Health Improvement, American Hospital Association, Chicago, IL, USA

**Keywords:** population health, undergraduates, training, workforce, curriculum, hospitals, health care reform

U.S. hospitals and health care systems are focusing increasing attention on health outcomes and the distribution of such outcomes as a means to improve the health of and to eliminate health inequities in their respective communities and patient populations. Much of this attention can be attributed to the provisions in the Patient Protection and Affordable Care Act (ACA) that explicitly promotes a population health approach by accelerating the transition to value-based payment models and by expanding access to health care services among newly insured Americans. As a result, hospitals and health care systems will need to realign their organizational infrastructures to be congruent with a population health management agenda. Health care leaders recognize that population health will be a key to their success moving forward, but identifying hospital-based leaders and training a workforce versed in population health will be critical to that success.

The way in which we train and properly prepare the health care sector workforce rest mainly with our for-profit and non-profit institutions of higher learning, baccalaureate nursing schools, other training programs, and the community college system where much of the important ancillary health care workforce first begin their professional training. Therefore, it begs the question: do our training and academic programs that lead to a career in the health care sector include a core public health component focusing specifically on population health competencies? If not, are we doing a disservice to the health care sector workforce, particularly the undergraduate trained individuals, by not preparing them for the demands their health care systems are now asking them to undertake in the realm of population health management? The workforce demand for undergraduates educated in public health is potentially in the thousands, and given the types and numbers of healthcare organizations, likely exceeds the demand for the direct public health workforce. For example, today the U.S. has 5,723 hospitals, over 33,000 medical group practices, 1,128 federally qualified health centers, and nearly 1,300 health insurance companies (1–5). Given the emphasis of the ACA on collaborative management of prospective and current patients, all of these organizations are likely to want staff knowledgeable about the parameters and metrics of population health. In the world of healthcare delivery, education for “public health” is synonymous with education for “population health.”

The significance of an undergraduate workforce educated about population health for the health care sector should not be minimized during this health system transformation. Many health care professionals trained at the undergraduate level will not have the opportunity to broaden their knowledge and expertise particularly in population health if they do not seek formal graduate education in public health. Prior to the implementation of the ACA, the reasons for this are many and this commentary will not try to list them all. However, one’s education on population health in their undergraduate training years may be impeded by: (1) their specific curriculum does not already have a built in public health component; (2) some health care training programs may not see a value in blending these competencies into their curriculum; (3) health care training programs are not listening to the needs of the employer community; and/or (4) health care employers are not demanding potential hires to already be trained in population health competencies.

In an attempt to better understand the best practices for population health as it relates to changing models of health care delivery and financing, the Association for Community Health Improvement (ACHI) and the American Hospital Association conducted an environmental scan among U.S. hospitals and health care systems on organizational structure, leadership, staffing, and community health initiatives. The information gathered and reported out is extremely insightful and further supports the continued need for a workforce educated about population health for the health care sector. All the findings will not be summarized here, but this commentary will highlight a particular segment of the hospital-based workforce to make the point that an undergraduate workforce educated about population health is vital.

ACHI has over 800 members, with representatives of more than 28% of the nation’s non-profit hospitals (1, 6). The 1,198 respondents completing a recent survey likely represent not only other ACHI members but also the many staff in other non-profit hospitals working on issues of community benefit and community outreach (7). Of note, almost half of the respondents (45%) whose principal responsibility is leading population health initiatives hold either bachelor’s degree, associate’s degree, or some other training (Figure 1). Further, the most important professional and educational backgrounds for staffing population health initiatives over the next few years were identified as community health, health education, and community benefit. Also, respondents identified community health needs assessments, healthy communities, collaborative facilitation, leadership, and community benefit as the most in-demand continuing professional education subjects (7). As hospitals adapt to using their community health needs assessments and community benefit requirement to advance their population health initiatives, their workforce needs will continue to evolve. Training institutions will need to prepare future hospital-based population health staff with a multidisciplinary background. More importantly, as an individual is determining their health care career focus during their undergraduate years, population health competencies should be a required interdisciplinary component of any health care undergraduate and associate degree curricula or other health care training programs. An education grounded in public health will be vital in providing a foundation for an individual interested in becoming an effective population health coordinator, manager, or leader for the health care sector no matter what role they hold inside the proverbial four walls.

**Figure 1:**
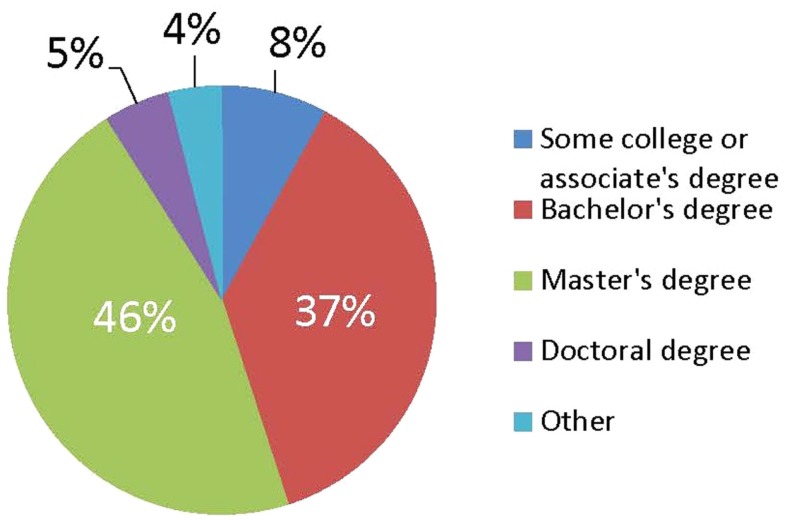
**Educational attainment of Person Heading Department with Principal Responsibility for Population Health (*n* = 1,148) (7)**.

Finally, population health knowledge and expertise have not historically been taught within most traditional American medical or health care curricula. Yet, hospital and health care system leaders recognize that advancing population health will enable them to thrive in a value-based landscape. Health care personnel needed now and in the foreseeable future for population health management will specifically need to be capable communicators and collaborative leaders who have the ability to understand and analyze the impacts of complex social systems on individual and community health, integrate public health concepts, use data to plan and evaluate their work, and possess those other skills acquired from the Core Competencies for Public Health Professionals (8). This demand creates a significant opportunity at the undergraduate level to start cultivating a workforce with the ability to integrate population health initiatives that are aligned with the community’s resources and needs well before they go on to advanced training or degrees in the health field.

## Conflict of Interest Statement

The author declares that the research was conducted in the absence of any commercial or financial relationships that could be construed as a potential conflict of interest.
